# Complete chloroplast genome sequence and comparative analysis of loblolly pine (*Pinus taeda* L.) with related species

**DOI:** 10.1371/journal.pone.0192966

**Published:** 2018-03-29

**Authors:** Sajjad Asaf, Abdul Latif Khan, Muhammad Aaqil Khan, Raheem Shahzad, Lubna, Sang Mo Kang, Ahmed Al-Harrasi, Ahmed Al-Rawahi, In-Jung Lee

**Affiliations:** 1 Chair of Oman’s Medicinal Plants & Marine Natural Products, University of Nizwa, Nizwa, Oman; 2 School of Applied Biosciences, Kyungpook National University, Daegu, Republic of Korea; 3 Department of Botany, Garden Campus, Abdul Wali Khan University Mardan, Mardan, Pakistan; 4 Research Institute for Dok-do and Ulleung-do Island, Kyungpook National University, Daegu, Republic of Korea; Montana State University Bozeman, UNITED STATES

## Abstract

Pinaceae, the largest family of conifers, has a diversified organization of chloroplast (cp) genomes with two typical highly reduced inverted repeats (IRs). In the current study, we determined the complete sequence of the cp genome of an economically and ecologically important conifer tree, the loblolly pine (*Pinus taeda* L.), using Illumina paired-end sequencing and compared the sequence with those of other pine species. The results revealed a genome size of 121,531 base pairs (bp) containing a pair of 830-bp IR regions, distinguished by a small single copy (42,258 bp) and large single copy (77,614 bp) region. The chloroplast genome of *P*. *taeda* encodes 120 genes, comprising 81 protein-coding genes, four ribosomal RNA genes, and 35 tRNA genes, with 151 randomly distributed microsatellites. Approximately 6 palindromic, 34 forward, and 22 tandem repeats were found in the *P*. *taeda* cp genome. Whole cp genome comparison with those of other *Pinus* species exhibited an overall high degree of sequence similarity, with some divergence in intergenic spacers. Higher and lower numbers of indels and single-nucleotide polymorphism substitutions were observed relative to *P*. *contorta* and *P*. *monophylla*, respectively. Phylogenomic analyses based on the complete genome sequence revealed that 60 shared genes generated trees with the same topologies, and *P*. *taeda* was closely related to *P*. *contorta* in the subgenus *Pinus*. Thus, the complete *P*. *taeda* genome provided valuable resources for population and evolutionary studies of gymnosperms and can be used to identify related species.

## Introduction

Gymnosperms are represented by a diverse and magnificent group of coniferous species distributed across eight families, consisting of 70 genera containing more than 630 species [1]. They are thought to have arisen from seed plants approximately 300 million years ago and are one of the ancient main plant clades. Gymnosperms possess larger genomes than flowering plants [2–5]. Recently, rapid progress has been made in angiosperm genome sequencing and analysis, but because of the complexity and order of magnitude increase in genome sizes, similar progress has not been attained for gymnosperms. Furthermore, comparative studies revealed that transposable elements, repetitive sequences, and gene duplication are common in gymnosperm genomes [4, 6–8]. Conifers are the main representatives of the gymnosperms, predominant in various ecosystems and representing 82% of terrestrial biomass [9].

*Pinus taeda* (loblolly pine) is a model species for the largest genus in the division Coniferae. It is an economically important and relatively fast-growing representative of conifers native to the southeastern United States. Previously, the loblolly pine was famous for providing pulp, lumber, and paper to commercial markets, but recently became a main bioenergy feedstock in lignocellulosic ethanol production [10]. Moreover, loblolly pine is considered an important species for comparative genomic studies between angiosperms and gymnosperms [8]. For example, microsatellites and single-nucleotide polymorphisms (SNPs) have been studied to determine population genetic parameters and the associations of phenotypes [11–13], create genetic maps [14–16], and develop genomic selection prediction models [17]. However, the number of available genetic markers remains small, particularly considering the large size of the pine genome. According to recent evaluations [18], the loblolly pine nuclear genome size is 21–24 Gbp. This is approximately four-fold larger than that of the angiosperm with the largest genome, *Hordeum vulgare* (barley), for which a reference genome is available, and approximately 7–8-fold larger than the human genome [19].

Chloroplasts are known to be derived from cyanobacterium through endosymbiosis and co-evaluation over time [20]. The gymnosperm chloroplast (cp) genome, particularly in conifers, has distinguishing characteristics among angiosperms. These features such as the high levels of variation (intra-specific) [21–24], paternal inheritance [25–28], and a different RNA editing pattern [29] were observed in studies. Generally, in angiosperms, cp genomes range from 130,000 to 160,000 base pairs (bp), with two duplicate inverted repeats (IRs) containing large single copy (LSC) and small single copy (SSC) regions. However, the comparative sizes of IRs, SSC, and LSC, are nearly unchanged, while the gene order and content are significantly conserved [30]. In contrast, the IR sizes of species form gymnosperms highly fluctuate among taxa [31–33]. Similarly, previous reports showed that the IR size for *Cycas taitungensis* is 23 kbp [34] and *Ginkgo biloba* is 17 kbp [35]. In contrast, *P*. *thunbergii* has a very small IR of 495 bp [36, 37]. Furthermore, in synergism with *P*. *thunbergii*, various conifer species have been found to lack the comparatively large IRs typically found in gymnosperms [31, 33, 38, 39]. This decrease in IR size is thought to cause extensive rearrangement in conifer cp genomes [33]. Based on the IRs, the cp genomes can be classified into three categories: (i) with two IRs, (ii) with one IRs, and (iii) with additional tandem repeats [30]. The cp genomes are essential and extremely valuable for understanding the phylogenetic relationships and designing specific molecular markers because of their firm mode of inheritance. Using a total evidence approach [40], the cp genomes or various concatenated sequences were studied to elucidate the phylogeny among various species [41–43]. Similarly, Steane [44] showed that the organization of the *P*. *thunbergii* cp genome differs from that of other related angiosperms.

The advent of high-throughput next-generation sequencing technologies from Illumina, Pacific Biosciences, Life Technologies, and Roche, among others, have rapidly improved genomic studies [45, 46]. In addition to draft or whole genomes of microbes and animals, genomic studies were performed to determine the chromosomal structures and molecular organization of wheat [47, 48] and maize [49]. In addition, these technologies have been extensively used to evaluate organelles, particularly chloroplast. Although the first complete nucleotide sequence of *Nicotiana tabacum* was generated by clone sequencing of plasmid and cosmid libraries over a long time [50], more than 800 cp genomes (including 300 from crops and trees) have now been sequenced and deposited in the NCBI Organelle Genome Resources database [51]. The evolution of cp genomes in terrestrial plants can now be studied using these database resources [51]. To date, a total of 16 complete chloroplast genomes in the genus *Pinus* have been sequenced and submitted to NCBI. In the current study, the complete cp genome of *P*. *taeda* (GenBank accession number: KY964286) was sequenced using next-generation sequencing tools. The goal of this study was to determine the cp genome organization of *P*. *taeda* and its global pattern of structural and comparative variation in the cp genome of *P*. *taeda* with 14 *Pinus* species (*P*. *koraiensis*, *P*. *sibirica*, *P*. *armandii*, *P*. *lambertiana*, *P*. *krempfii*, *P*. *bungeana*, *P*. *gerardiana*, *P*. *monophylla*, *P*. *nelsonii*, *P*. *contorta*, *P*. *massoniana*, *P*. *tabuliformis*, *P*. *taiwanensis*, *P*. *strobus*, and *P*. *thunbergii*).

## Materials and methods

### Chloroplast genome sequencing and assembly

Plastid DNA was extracted from the fresh needle leaf parts of *P*. *taeda* using the DNeasy Plant Mini Kit (Qiagen, Hilden, Germany), and the resulting cpDNA was sequenced using an Illumina HiSeq-2000 platform (San Diego, CA, USA) at Macrogen (Seoul, Korea). The *P*. *taeda* cp genome was then assembled *de novo* using a bioinformatics pipeline (http://www.phyzen.com). Specifically, a 400-bp paired-end library was produced according to the Illumina standard method, which generated 28,110,596 bp of sequence data with a 100-bp average read length. Raw reads with Phred scores of ≤20 were removed from the total PE reads using the CLC-quality trim tool, and *de novo* assembly of trimmed reads was accomplished using CLC Genomics Workbench v7.0 (CLC Bio, Aarhus, Denmark) with a minimum overlap of 200–600 bp. The resulting contigs were compared against the *P*. *thunbergii* and *P*. *contorta* plastomes using BLASTN with an E-value cutoff of 1e-5, and five contigs were identified and temporarily arranged based on their mapping positions on the reference genome. After initial assembly, primers were designed (S1 Table) based on the terminal sequences of adjacent contigs, and PCR amplification and subsequent DNA sequencing were conducted to fill in the gaps. PCR amplification was performed in 20-μL reactions containing 1× reaction buffer, 0.4 μL dNTPs (10 mM), 0.1 μL Taq (Solg h-Taq DNA Polymerase), 1 μL (10 pm/μL) primers, and 1 μL (10 ng/μL) DNA, using the following conditions: initial denaturation at 95°C for 5 min; 32 cycles of 95°C for 30 s, 60°C for 20 s, and 72°C for 30 s; and a final extension step of 72°C for 5 min. After incorporating the additional sequencing results, the complete cp genome was used as a reference to map the remaining unmapped short reads to improve the sequence coverage of the assembled genome.

### Analysis of gene content and sequence architecture

The *P*. *taeda* cp genome was annotated using DOGMA [52], checked manually, and the codon positions were adjusted by comparison with homologs in the cp genome of *P*. *taeda* and *P*. *contorta*. Transfer RNA sequences of the *P*. *taeda* cp genome were verified using tRNAscan-SE version 1.21 [53] with default settings, and the structural features were illustrated using OGDRAW [54]. To examine deviations in synonymous codon usage by avoiding the influence of amino acid composition, the relative synonymous codon usage was determined using MEGA 6 software [55], and finally the divergence of the *P*. *taeda* cp genome from six other *Pinus* species (five from subgenus *Pinus* and one from subgenus *Strobus*) cp genomes was assessed using mVISTA [56] in Shuffle-LAGAN mode and using the *P*. *taeda* genome as a reference.

### Elucidation of repeat sequences and simple sequence repeat (SSRs)

Repeat sequences, including direct, reverse, and palindromic repeats, were identified within the cp genome using REPuter [57] with the following settings: Hamming distance of 3, ≥90% sequence identity, and minimum repeat size of 30 bp. Furthermore, SSRs were detected using Phobos version 3.3.12 [58] with the search parameters set to ≥10 repeat units for mononucleotide repeats, ≥8 repeat units for dinucleotide repeats, ≥4 repeat units for trinucleotide and tetranucleotide repeats, and ≥3 repeat units for pentanucleotide and hexanucleotide repeats. Tandem repeats were identified using Tandem Repeats Finder version 4.07 b [59] with default settings.

### Sequence divergence and phylogenetic analyses

The average pairwise sequence divergence of 60 shared genes and complete plastomes of 15 *Pinus* species was analyzed, using data from *P*. *taeda*, *P*. *koraiensis*, *P*. *sibirica*, *P*. *armandii*, *P*. *lambertiana*, *P*. *krempfii*, *P*. *bungeana*, *P*. *gerardiana*, *P*. *monophylla*, *P*. *nelsonii*, *P*. *contorta*, *P*. *massoniana*, *P*. *tabuliformis*, *P*. *taiwanensis*, *P*. *strobus*, and *P*. *thunbergii*. In cases of missed and unclear genes, annotation was confirmed by comparison with the reference sequence after assembling a multiple sequence alignment tool. The complete genome data set was aligned using MAFFT version 7.222 [60] with default parameters. For pairwise sequence divergence, a Kimura’s model was used [61]. Indel polymorphisms among the complete genomes were identified using DnaSP 5.10.01 [62], and a custom Python script (https://www.biostars.org/p/119214/) was used to identify SNPs. To resolve the phylogenetic position of *P*. *taeda* within the genus *Pinus*, 14 published *Pinus* species plastomes were downloaded from the NCBI database for phylogenetic analysis. Multiple alignments of the complete plastomes were constructed based on the conserved structure and gene order of the plastid genomes [63], and four methods were employed to construct phylogenetic trees, including Bayesian inference (BI), which was implemented using MrBayes 3.1.2 [64], maximum parsimony (MP), which was implemented using PAUP 4.0 [65], and maximum likelihood (ML) and neighbor-joining (NJ), which were implemented using MEGA 6 [55] using previously described settings [66, 67]. In a second phylogenetic analysis, 60 shared cp genes from 15 *Pinus* species, including *P*. *taeda*, and one outgroup species (*Juniperus bermudiana*) were aligned using ClustalX with default settings, followed by manual adjustment to preserve the reading frames. Finally, the same four phylogenetic inference methods were used to infer trees from the 60 concatenated genes using the same settings [66, 67].

## Results and discussion

**Fig 1:**
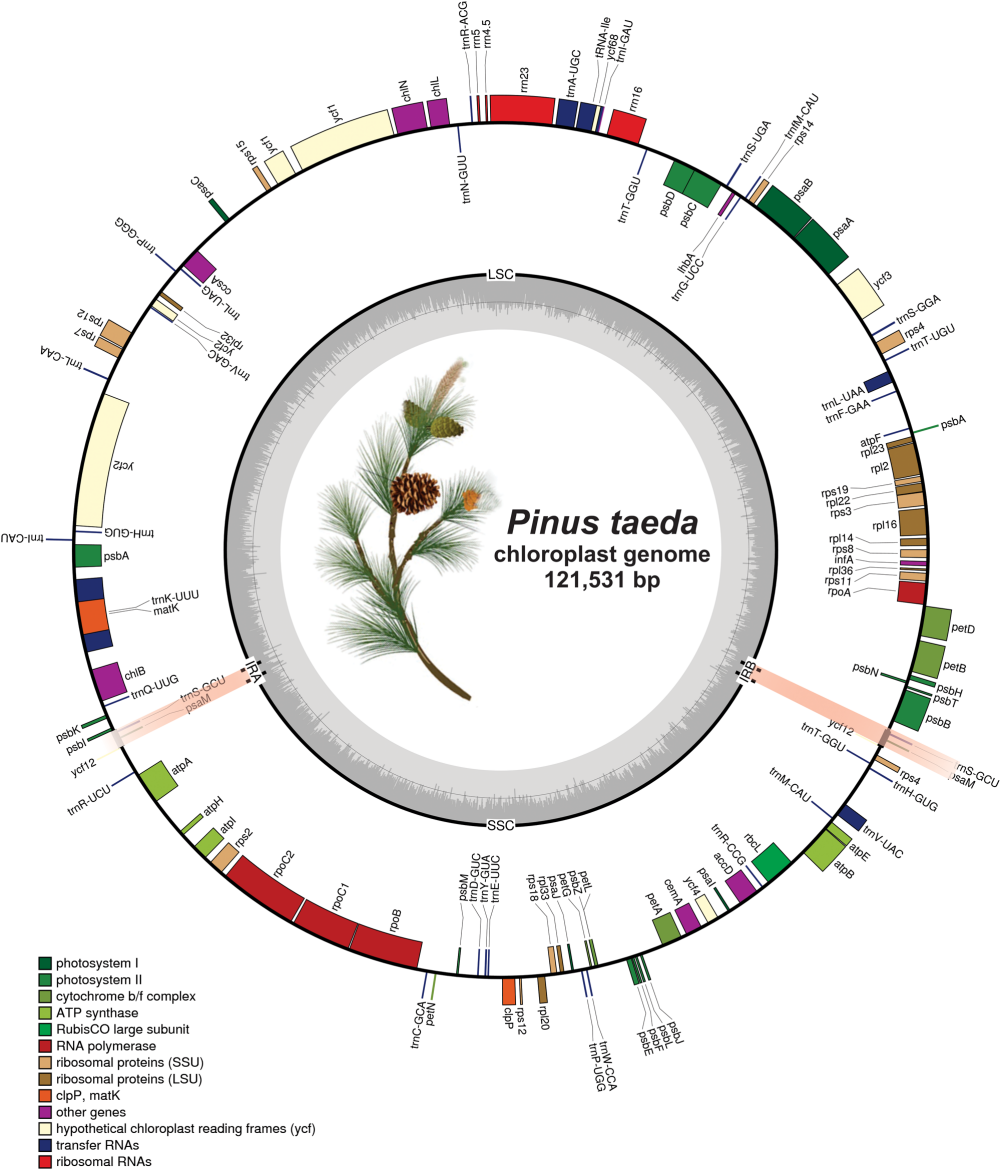
Gene map of the *Pinus taeda* plastid genome. Thick lines in the red area indicate the extent of the inverted repeat regions (IRa and IRb; 850 bp), which separate the genome into small (SSC; 42,258 bp) and large (LSC; 77,614 bp) single copy regions. Genes drawn inside the circle are transcribed clockwise, and those outside are transcribed counter clockwise. Genes belonging to different functional groups are color-coded. The dark grey in the inner circle corresponds to the GC content and the light grey corresponds to the AT content.

The *P*. *taeda* cp genome was assembled by mapping all Illumina sequence reads into a draft cp genome. Approximately 2,5131,617 reads with 100-bp average lengths were retrieved to obtain 1619.4X coverage of the cp genome. The complete cp genome of *P*. *taeda* was 121,131 bp, with 38.5% GC content and only one bp less than the previously sequenced *P*. *taeda* cp genome (Table 1). The cp genome size of *P*. *taeda* was within the expected range (116–121 Kb) of other sequenced cp genomes of Pinaceae members [41, 68, 69]. The *P*. *taeda* cp genome was circular and contained two short-inverted repeats (IRa and IRb) of 830 bp, divided into SSC (42,258 bp) and LSC (77,614 bp) (Fig 1). The *P*. *taeda* cp genome encodes 120 genes, including 81 protein-coding genes, four ribosomal RNA (rRNA) genes, and 35 tRNA genes (Table 2). Of these genes, 11 genes (*atpF*, *petB*, *petD*, *rpoC1*, *rpl2*, *rpl16*, *trnI-GAU*, *trnG-UCC*, *trnA-UGC*, *trnV-UAC*, and *trnL-UAA*) contained one intron and two genes (*rps12* and *ycf3*) harbored two introns (Table 3). Furthermore, *trnK-UUU* was identified as the gene containing the longest intron (3,307 bp), which included *matK* (Table 3); similarly, *rps12* was recognized as a trans-spliced gene, with the N-terminal exon-I located at 92 Kb from C-terminal exons-II and III as reported previously for various gymnosperms [70].

**Table 1:** Summary of complete chloroplast genomes for 15 *Pinus* species.

	*P*. *tae*	*P*.tae*	*P*.*arm*	*P*. *bung*	*P*. *cont*	*P*. *gerar*	*P*. *kor*	*P*. *krem*	*P*. *lamb*	*P*. *mass*	*P*. *mono*	*P*. *nel*	*P*. *sib*	*P*. *tab*	*P*. *taiw*	*P*. *stro*	*P*. *thu*
**Size (bp)**	121,531	121,530	117,265	117,861	120,438	117,618	117,190	116,989	117,239	119,739	116,479	116,834	116,635	119,646	119,741	115,576	119,707
**Overall GC contents**	38.5	38.5	38.8	38.1	38.4	38.7	38.8	38.7	38.7	38.5	38.6	-	38.7	38.5	38.5	38.8	38.5
**LSC size in bp**	77,614	77,615	64,548	65,373	59,591	-	64,523	-	64,750	51,458	74,357	-	64,080	75,628	65,670	74,634	65,696
**SSC size in bp**	42,258	42,532	51,767	51,538	60,131	-	51,717	-	51,715	43,197	41,691	-	51,782	42,329	53,080	40,310	53,020
**IR size in bp**	830	693	475	475	358	-	475	-	387	378	431	-	387	845	409	467	495
**Protein coding regions size in bp**	61,691	60,765	61,227	60,702	58,469	60,364	60,496	59,753	60,847	60,519	60,015	69,598	62,988	60,549	65,133	53,919	70,395
**tRNA size in bp**	2,661	2,587	2,778	2,725	2,582	2,583	2,778	2,428	2,511	2,725	2,577	2,575	2,131	2,725	2,785	2,657	2,652
**rRNA size in bp**	4,517	4,517	4,555	4,515	4,517	4,515	4,555	4,514	4,515	4,515	4,515	4,515	4,555	4,518	4,518	4,516	4,518
**Number of genes**	122	111	115	113	110	110	110	108	110	109	111	111	113	116	137	111	171
**Number of protein coding genes**	83	71	74	71	70	70	70	69	71	73	70	70	81	74	92	70	123
**Number of rRNA**	4	4	4	4	4	4	4	4	4	4	4	4	4	4	4	4	4
**Number of tRNA**	35	34	36	36	34	34	36	32	33	36	34	34	28	36	36	35	35
**Genes duplicated in IR**	3	2	2	2	1		4		1	1	1		1	3	1	1	2
**Genes with introns**	13	13	13	14	13	13	15	13	13	15	13	13	13	14	13	13	15

**P. tae** = *P*. *taeda*; P. tae* = *P*. *taeda* (old); **P.arm** = *P*. *armandii*; **P. bung** = *P*. *bungeana*; **P. cont** = *P*. *contorta*; **P. gerar** = *P*. *gerardiana*; **P. kor** = *P*. *koraiensis*; **P. krem** = *P*. *krempfii*; **P. lamb** = *P*. *lambertiana*; **P. mass** = *P*. *massoniana*; **P. mono** = *P*. *monophylla*; **P. nel** = *P*. *nelsonii*; **P. sib** = *P*. *sibirica*; **P. tab** = *P*. *tabuliformis*; **P. taiw** = *P*. *taiwanensis*; **P. stro** = *P*. *strobus*; **P. thu** = *P*. *thunbergii*

**Table 2:** Genes in the sequenced *P*. *taeda* chloroplast genome.

Category	Group of genes	Name of genes
**Self-replication**	Large subunit of ribosomal proteins	*rpl2*, *14*, *16*, *20*, *22*, *23*, *32*, *33*, *36*
	Small subunit of ribosomal proteins	*rps2*, *3*, *4*, *7*, *8*, *11*, *12*, *14*, *15*, *18*, *19*
	DNA-dependent RNA polymerase	*rpoA*, *B*, *C1*, *C2*
	rRNA genes	*RNA*
	tRNA genes	*trnA-UGC*, *trnC-GCA*, *trnD-GUC*, *trnE-UUC*, *trnF-GAA*, *trnfM-CAU*, *trnG-UCC*, *trnH-GUG*, *trnI-CAU*, *trnI-GAU*, *trnK-UUU*, *trnL-CAA*, *trnL-UAA*, *trnL-UAG*, *trnM-CAU*, *trnN-GUU*, *trnP-GGG*, *trnP-UGG*, *trnQ-UUG*, *trnR-ACG*, *trnR-UCU*, *trnS-GCU*, *trnS-GGA*, *trnS-UGA*, *trnT-GGU*, *trnT-UGU*, *trnV-GAC*, *trnV-UAC*, *trnW-CCA*, *trnY-GUA*
**Photosynthesis**	Photosystem I	*psaA*, *B*, *C*, *I*, *J*, *M*
	Photosystem II	*psbA*, *B*, *C*, *D*, *E*, *F*, *H*, *I*, *J*, *K*, *L*, *M*, *N*, *T*, *Z*
	Cytochrome b6/f complex	*petA*, *B*, *D*, *G*, *L*, *N*
	ATP synthase	*atpA*, *B*, *E*, *F*, *H*, *I*
	Rubisco	*rbcL*
	Chlorophyll biosynthesis	*chlB*, *L*, *N*
**Other genes**
	Maturase	*matK*
	Protease	*clpP*
	Envelop membrane protein	*cemA*
	Subunit acetyl-CoA-carboxylate	*accD*
	c-Type cytochrome synthesis gene	*ccsA*
**Unknown**	Conserved open reading frames	*ycf1*, *2*, *3*, *4*, *12*, *68*

**Table 3:** Genes with introns in the *Pinus taeda* chloroplast genome and length of exons and introns.

Gene	Location	Exon I (bp)	Intron 1 (bp)	Exon II (bp)	Intron II (bp)	Exon III (bp)
*atpF*	LSC	159	740	408		
*petB*	LSC	6	799	648		
*petD*	LSC	8	698	667		
*rpl2*	IR	402	668	429		
*rpl16*	LSC	9	835	396		
*rpoC1*	LSC	432	674	1665		
*rps12*		114	-	232	540	26
*ycf3*	LSC	124	726	230	709	156
*trnA-UGC*	IR	38	770	35		
*trnI-GAU*	IR	42	974	35		
*trnL-UAA*	LSC	50	488	35		
*trnK-UUU*	LSC	35	3307	37		
*trnV-UAC*	LSC	39	541	37		

**Fig 2:**
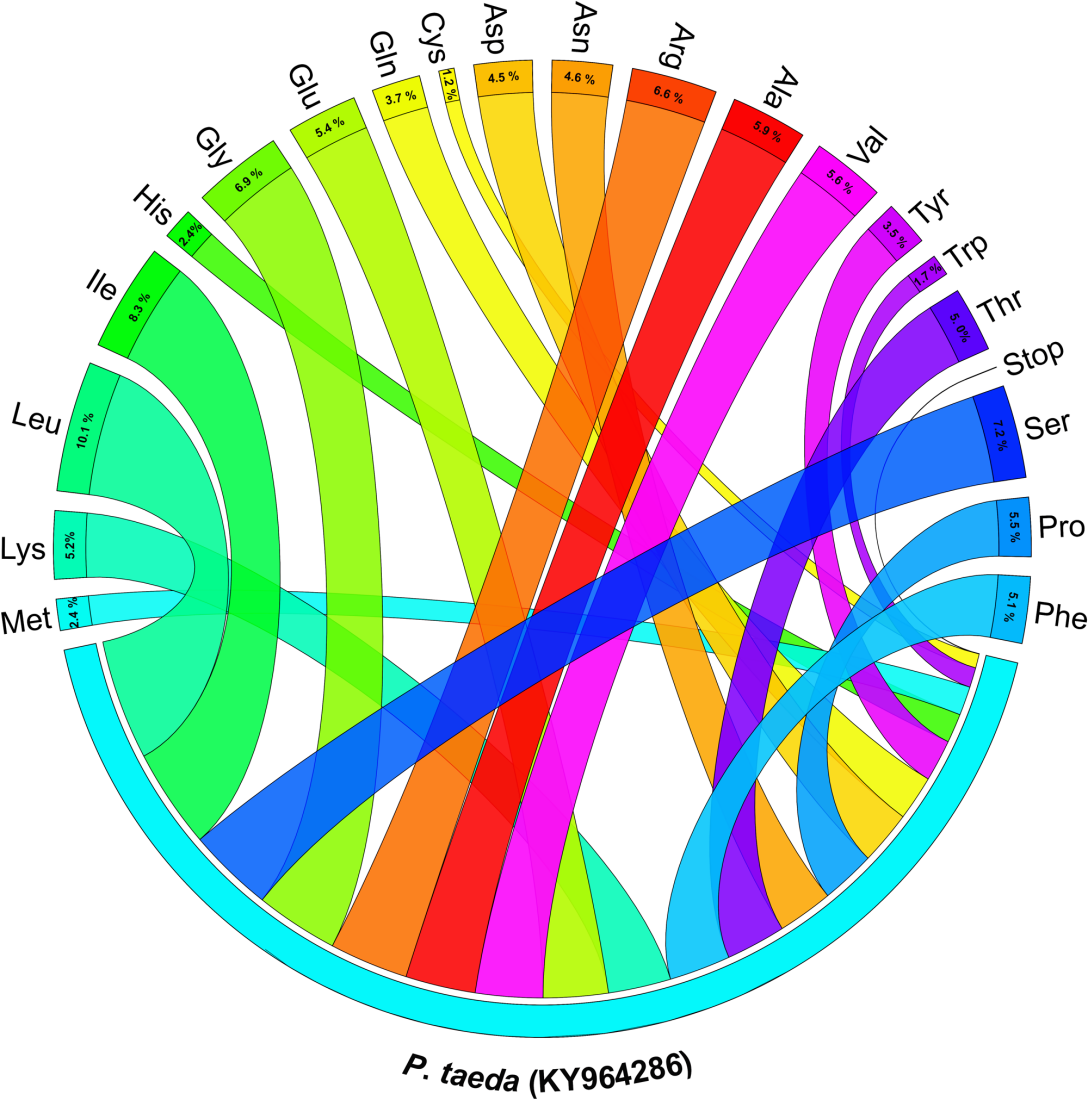
Amino acid frequencies of the *Pinus taeda* chloroplast (cp) protein coding sequences. The frequencies of amino acids were calculated for all 81 protein-coding genes from the start to the stop codon.

The protein coding regions containing 81 genes were 61,691 bp and accounted for 50.76% of the *P*. *taeda* cp genome. In the *P*. *taeda* cp genome, the gene proportion for tRNA was 2.18% and for rRNA it was 3.71%. A total of 43.35% of the non-coding region was composed of introns and intergenic spacers. The total protein-coding sequences encoded 20,563 codons (Table 4). The codon-usage frequency was calculated based on protein-coding and tRNA gene sequences (Table 5). Leucine was the most coded (2,067, 10.1%) and cysteine was the least coded (244, 1.2%) amino acid (Fig 2). Similar ratios for amino acids were found in previously reported cp genomes [71, 72]. The maximum GAA (835; 4.06%) and minimum TGC (65; 0.316%) codons used coded for glutamic acid and encoding cysteine, respectively. The A-T content was 50.6%, 59.99%, and 69.97% at the three consecutive codon positions (Table 4). The preference for the high A-T content at the 3^rd^ codon position is similar to the A and T concentrations reported in various terrestrial plant cp genomes [72–74].

**Table 4:** Base compositions in the *Pinus taeda* chloroplast (cp) genome.

	T/U	C	A	G	Length (bp)
**Genome**	30.8	19.3	30.7	19.3	121,531
**LSC**	30.7	19.0	30.3	20.0	77,614
**SSC**	31.3	19.5	31.0	18.3	42,258
**IR**	31.1	20.2	31.1	17.6	830
**tRNA**	23.7	24.9	22.4	29.0	2661
**rRNA**	18.8	23.6	26.4	31.1	4517
**Protein coding genes**	30.5	18.1	30.5	20.9	61,691
**1st position**	20.4	16.03	30.26	28.3	20,563
**2nd position**	31.5	20.7	28.49	18.2	20,563
**3rd position**	38.18	13.94	31.79	16.07	20,563

**Table 5:** Codon–anticodon recognition pattern and codon usage for the *Pinus taeda* chloroplast genome.

Amino acid	Codon	No	RSCU	tRNA	Amino acid	Codon	No	RSCU	tRNA
Phe	UUU	1394	1.11		Tyr	UAC	562	0.66	*trnY-GUA*
Phe	UUC	1108	0.89	*trnF-GAA*	Tyr	UAU	1137	1.34	
Leu	UUA	841	1.23	*trnL-UAA*	Stop	UAA	776	1.05	
Leu	UUG	815	1.19	*trnL-CAA*	Stop	UGA	781	1.06	
Leu	CUU	818	1.2		Stop	UAG	662	0.89	
Leu	CUC	533	0.78		Cyc	UGC	378	0.9	*trnC-GCA*
Leu	CUA	642	0.94	*trnL-UAG*	Trp	UGG	677	1	*trnW-CCA*
Leu	CUG	444	0.65		His	CAU	839	1.43	
Ile	AUU	1233	1.09		His	CAC	337	0.57	*trnH-GUG*
Ile	AUC	963	0.85	*trnI-GAU*	Gln	CAA	842	1.27	*trnQ-UUG*
Ile	AUA	1194	1.06	*trnI-CAU*	Gln	CAG	481	0.73	
Met	AUG	807	1	*trn(f)M-CAU*	Asn	AAU	1318	1.34	
Val	GUU	652	1.29		Asn	AAC	644	0.66	*trnN-GUU*
Val	GUC	365	0.72	*trnV-GAC*	Lys	AAA	1444	1.3	*trnK-UUU*
Val	GUA	606	1.2	*trnV-UAC*	Lys	AAG	770	0.7	
Val	GUG	391	0.78		Asp	GAU	917	1.43	
Ser	UCC	752	1.22	*trnS-GGA*	Asp	GAC	368	0.57	*trnD-GUC*
Ser	UCA	767	1.25	*trnS-UGA*	Glu	GAA	1043	1.33	*trnE-UUC*
Ser	UCG	431	0.7		Glu	GAG	529	0.67	
Pro	CCU	516	1.11		Arg	CGU	278	0.67	*trnR-ACG*
Pro	CCC	400	0.86	*trnP-GGG*	Arg	CGC	163	0.39	
Pro	CCA	624	1.35	*trnP-UGG*	Arg	CGA	439	1.06	
Pro	CCG	313	0.68		Arg	CGG	284	0.68	
Thr	ACU	448	1.05		Ser	AGU	499	0.81	
Thr	ACC	497	1.17		Ser	AGC	387	0.63	*trnS-GCU*
Thr	ACA	441	1.03	*trnT-UGU*	Arg	AGA	821	1.97	*trnR-UCU*
Thr	ACG	320	0.75		Arg	AGG	511	1.23	
Ala	GCU	397	1.38		Gly	GGU	456	0.99	
Ala	GCC	233	0.81		Gly	GGC	214	0.46	*trnG-GCC*
Ala	GCA	347	1.21	*trnA-UGC*	Gly	GGA	728	1.57	*trnG-UCC*
Ala	GCG	172	0.6		Gly	GGG	451	0.98	

### Difference in gene contents of *P*. *taeda*

**Fig 3:**
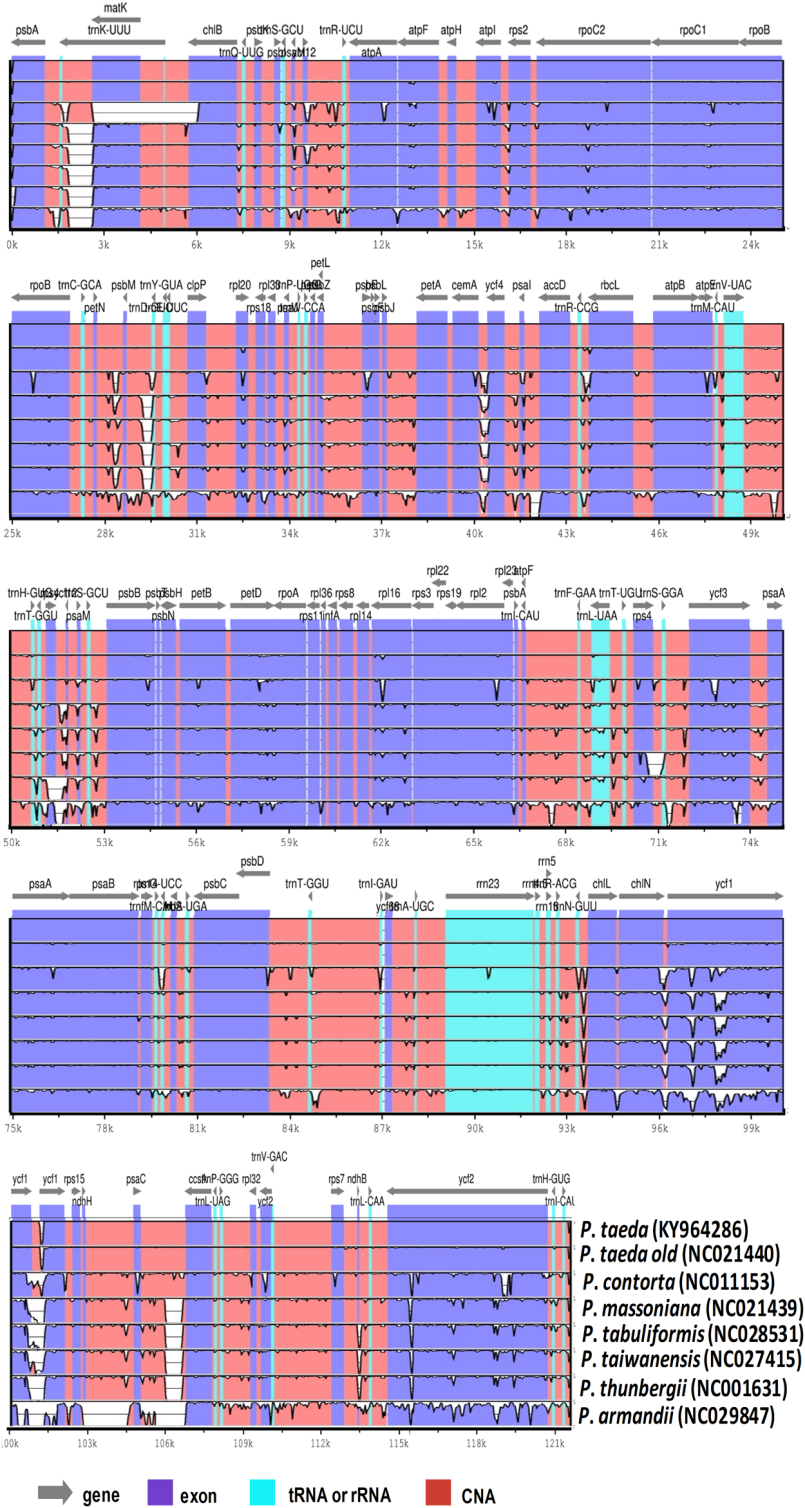
Visual alignment of plastid genomes from *Pinus taeda* and six other *Pinus* species (five from the subgenus *Pinus* and one from the subgenus *Strobus*). VISTA-based identity plot showing sequence identity among seven species, using *P*. *taeda* as a reference.

We selected 16 cp genomes in the *Pinus* genus (*P*. *taeda* (old), *P*. *koraiensis*, *P*. *sibirica*, *P*. *armandii*, *P*. *lambertiana*, *P*. *krempfii*, *P*. *bungeana*, *P*. *gerardiana*, *P*. *monophylla*, *P*. *nelsonii*, *P*. *contorta*, *P*. *massoniana*, *P*. *tabuliformis*, *P*. *taiwanensis*, *P*. *strobus*, and *P*. *thunbergii*) for comparison with *P*. *taeda* (new) (121,531 bp). *Pinus taeda* had the largest genome. The differentiation can be ascribed to the variation in size of LSC (Table 1). Analysis of known genes functions revealed that *P*. *taeda* shared 60 different protein-coding genes with 15 other *Pinus* species. Furthermore, pairwise alignment between the cp genome of *P*. *taeda* and six related cp genomes showed the highest synteny. Annotation of the *P*. *taeda* cp genome was used for plotting the total sequence identity of the six cp genomes of *Pinus* species in mVISTA (Fig 3). The results revealed high sequence identity with five species from the subgenus *Pinus* (*P*. *contorta*, *P*. *massoniana*, *P*. *tabuliformis*, *P*. *taiwanensis*, and *P*. *thunbergii*) compared to *P*. *armandii* from the subgenus *Strobus*. However, for all species, relatively lower identity was observed in various comparable genomic regions, particularly the *trnK-UUU*, *matK*, *atpI*, *rpl16*, *petB*, *petD*, *ycf1*, and *ycf2* regions (Fig 3). Similarly, non-coding regions exhibited greater bifurcation than the coding-regions. Among the diverging regions, *psbA*-*chlB*, *psbM*- *clpP*, *ycf4-accD*, *ycf3- psaA*, *psaC-ccsA*, *ndhH- psaC*, *ycf3-psaA*, *trnG-UUU- chlL*, and *petL- psbF* were significant. The current findings agree with the results previously reported for these genes in angiosperm cp genomes [43, 72]. Our results confirmed similar variations among the coding-regions of the investigated species. This was also suggested by Kumar et al. [75]. Furthermore, comparison of the *P*. *taeda* whole cp genome with those of related species revealed lower SNP and indel substitutions for the subgenus *Pinus* cp genomes, which ranged from 809 in *P*. *taeda* (old) to 2,636 in *P*. *thunbergii*. However, the results revealed higher SNP and indel substitutions within the subgenus *Strobus* cp genomes, which ranged from 9,211 in *P*. *gerardiana* to 19,196 in *P*. *monophylla* (S2 Table). These results indicate the presence of interspecific mutations in the highly conservative cp genome that may be useful for analyzing genetic diversity and evolution. Similarly, we evaluated pairwise-sequence differentiation among the 16 pine species (S3 Table). The results showed that the *P*. *taeda* genome had 0.0274 average sequence divergences, high divergence was detected for *P*. *nelsonii* (0.0402), and *P*. *taeda* (old) had the lowest average sequence divergence (0.00321) followed by *P*. *contorta* (0.00807).

The gene organization and gene contents of the cp genomes are generally conserved compared with those in the mitochondrial and nuclear genomes [76]. The cp genome organization and structure are extremely conserved in angiosperms, i.e. there is a distinctive quadripartite structure containing an SSC region and LSC region separated by a pair of inverted repeats [77]. In contrast, various genome rearrangements have been detected in various gymnosperms cp genomes [78, 79]. While the *P*. *taeda* cp genome shared some similar characteristics with other plants, we detected noticeable differentiation in numerous genes among gymnosperms. For example, significant divergence was noted in the gene content between *P*. *taeda* and other gymnosperms. For instance, in *Cryptomeria japonica*, eleven intact NADH dehydrogenase genes were identified, which were correlated to 5 other plant species [37], but were not present in the *P*. *taeda* and *P*. *thunbergii* cp genomes [37]. Previously, it was reported that the loss of NADH dehydrogenases was caused by specific mutations in the cp genome of *Pinus* [79].

In contrast, an essential gene, *rps16*, was completely absent from the *P*. *taeda* cp genome. Similar results were reported for the *P*. *thunbergii* and *Marchantia polymorpha* [36, 80] cp genomes, in addition to various terrestrial plants species, including *Eucommia*, *Epifagus*, *Fugus*, *Malpighia*, *Krameria*, *Passiflora*, *Connarus*, *Linum*, *Turnera*, *Securidaca*, *Medicago*, *Selaginella*, *Viola*, and *Adonis* [81–86]. In contrast, *rps16* is present in the angiosperms *Oryza sativa* and *E*. *globulus*, in the fern *Adiantum capillus*, and in the gymnosperms *C*. *japonica* and *C*. *taitungensis*. However, the position of *rps16* is different in gymnosperms from that in angiosperm cp genomes. The position is intermediate between *chlB* and *trnK-UUU* in the gymnosperm cp genomes and halfway between *trnQ-UUG* and *trnK-UUU* and between *chlB* and *matK* in angiosperms and ferns, respectively. Doyle *et al*. [83] suggested the functional transfer of *rps16* to the nucleus from chloroplasts and the absence of this gene from various terrestrial plants. Furthermore, it was reported that the loss of *rps16* and its functional transfer to the nucleus may have occurred autonomously in gymnosperms, particularly in coniferous species.

*trnR-CCG* and *trnP-GGG* are also found in *P*. *taeda* cp genomes. These genes are reported as pseudo genes and are likely relics of cp genome evolution in mosses and gymnosperms [29, 87, 88]. *trnP-GGG* was previously reported in two gymnosperms, *C*. *taitungensis* and *P*. *thunbergii*, as well as in *C*. *japonica*, in the fern *A*. *capillus* and liverwort *M*. *polymorpha*, and but was absent from the cp genomes of angiosperms. This gene was also identified in *Ginkgo* and *Gnetum* [34], revealing that the gene is common in numerous gymnosperm species. Similarly, *trnR-CCG* in *P*. *taeda* was previously reported in *C*. *taitungensis*, *A*. *capillus*, *P*. *thunbergii*, and *M*. *polymorpha*. However, the absence of this gene in *C*. *japonica* and various cp genomes of angiosperms suggests that *trnR-CCG* is not well-maintained in the cp genomes of all gymnosperms and may have been lost in various taxa during plant evolution [79].

Furthermore, *clpP*, which encodes a proteolytic subunit of the ATP-dependent *clpP* protease, contains no intron in the *P*. *taeda* cp genome. Similar results were previously reported for *P*. *thunbergii*, *P*. *mugo*, *P*. *dabeshanensis*, and *P*. *taiwanensis* [37, 41, 68, 89]. In contrast, *clpP* is found in the cp genome of other land plants, such as *A*. *capillus*, *E*. *globulus*, *M*. *polymorpha*, and *C*. *taitungensis* with two or three exons [29]. However, in the *P*. *taeda* cp genome, only the *clpP* second exon remained, and as such, it occurs as a pseudogene. Similarly, the *rpl20 and clpP* order is conserved in the *P*. *taeda* cp genome and *clpP* is co-transcribed with the 5’-end of *rps12* and *rpl20*, as reported previously for the cp genomes of various gymnosperms [90, 91] [92]. *accD* encodes acetyl-CoA-carboxylase and has been found in the *P*. *taeda* cp genome. The reading frame length of *accD* was similar to that of the cp genomes of other Pinaceae members and has 321 codons, which is fewer than that in *C*. *japonica* (700 codons) and more than the 309 codons of *A*. *capillus* and 316 codons of *M*. *polymorpha*. Furthermore, in angiosperms, particularly monocots, the reading-frame size of *accD* has been reduced from 106 codons in *Oryza sativa* to none in *Zea mays*. This has also been suggested as reason for the loss of *accD* in monocot plant species [93]. In contrast, the *accD* reading-frame in gymnosperms, particularly in coniferous species and *C*. *japonica*, may have diverted in the ascending direction.

### Loss of large IR region within the *P*. *taeda* cp genome

**Fig 4:**
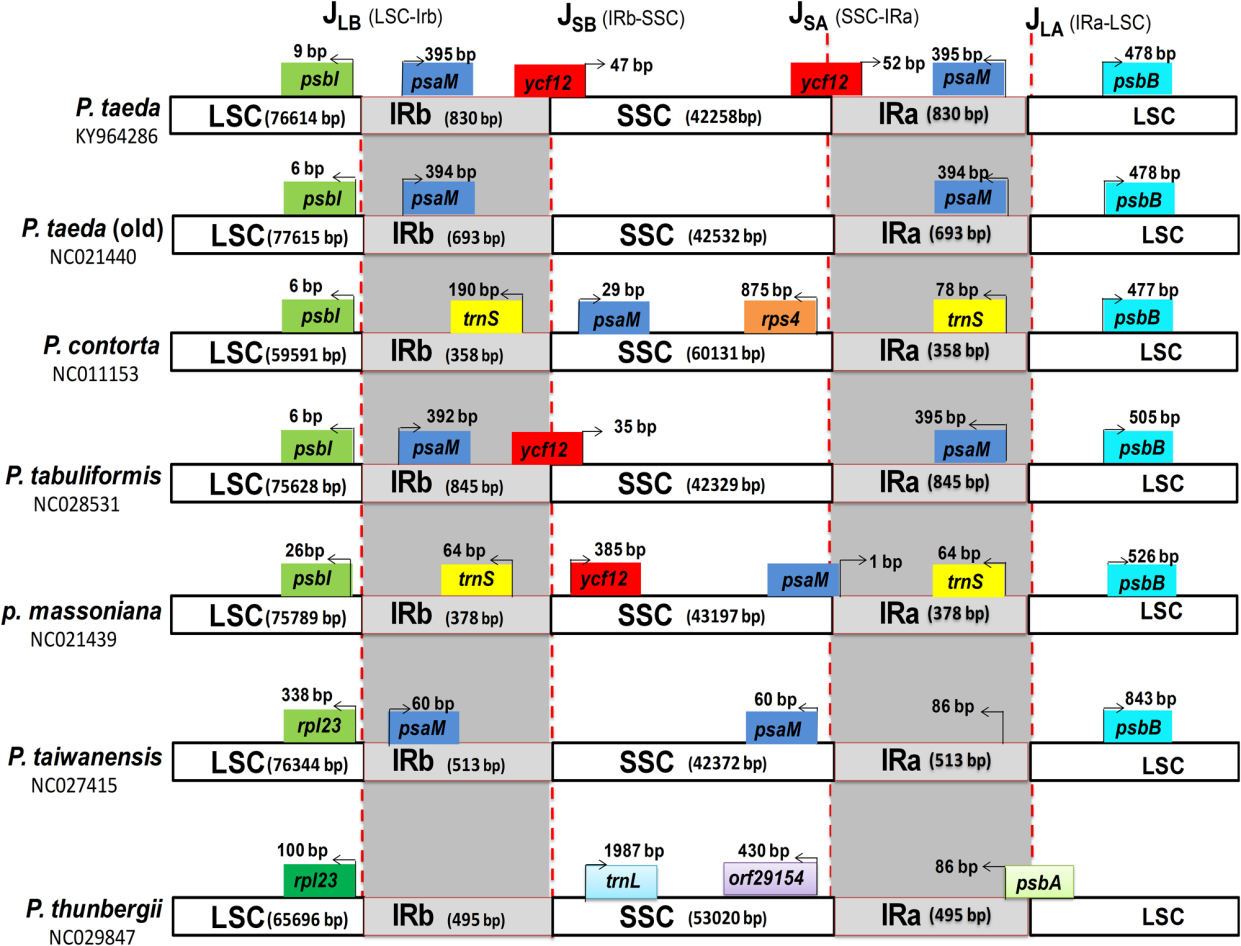
Distance between adjacent genes and junctions of the small single-copy (SSC), large single-copy (LSC), and two inverted repeat (IR) regions among plastid genomes from six *Pinus* species. Boxes above and below the main line indicate the adjacent border genes. The figure is not to scale regarding sequence length, and only shows relative changes at or near the IR/SC borders.

The large inverted repeat regions, which have been reported in various land plant cp genomes, were reduced to two very short inverted repeat (IRa and IRb) regions of 830 bp in *P*. *taeda*, and were separated by a SSC region of 42,258 bp and LSC region of 77,614 bp (Fig 1). However, in the previously sequenced *P*. *taeda* cp genome submitted to NCBI, the short inverted repeat regions were 693 bp (Table 1). Similar results were observed in other Pinaceae members, such as *P*. *taiwanensis*, *P*. *armandii*, and *P*. *dabeshanensis*, where the inverted repeat sizes were reduced to 513, 475, and 473 bp, respectively [68, 69, 89]. The IR of *P*. *taeda* contained duplicated *psaM* and *trnS-GCU* and partial *ycf12*, apparently caused by incomplete loss of the large IR, as reported previously for various gymnosperms [36, 37]. Detailed comparison of four junctions (J_LA_, J_LB_, J_SA_, and J_SB_) between the two IRs (IRa and IRb) and two single-copy regions (LSC and SSC) was performed between *Pinus* species (*P*. *contorta*, *P*. *tabuliformis*, *P*. *massoniana*, *P*. *taiwanensis*, and *P*. *thunbergii*) and *P*. *taeda* by carefully analyzing the exact IR border positions and adjacent genes (Fig 4). Some IR expansion and contraction were observed in the *P*. *taeda* cp genome compared to that of the other five *Pinus* species, which ranged from 358 bp (*P*. *contorta*) to 845 bp (*P*. *tabuliformis*) (Fig 4). The genes marking the beginning and end of the IRs were only partially duplicated. *psbI* in *P*. *taeda* was located 9 bp from J_LB_ in the LSC region. In *P*. *contorta*, *P*. *tabuliformis*, and *P*. *taeda* (old), this distance was 6 bp, whereas in *P*. *massoniana* and *P*. *taiwanensis* the distances were 26 and 338 bp, respectively. However, variation was found in *P*. *thunbergii*, and *rpl23* was 100 bp away from J_LB_ in the LSC region. Similarly, hypothetical chloroplast *ycf12* was partially duplicated by 47 bp (*P*. *taeda*) and 35 bp in *P*. *tabuliformis*. However, in *P*. *massoniana*, *ycf12* was located in the SSC region, 385 bp away from J_SB_. In *P*. t*aeda* and *P*. *tabuliformis*, J_LA_ was located between *psaM* and *psbB* and the difference in distance between *psaM* and J_LA_ was 395 bp. However, in *P*. *contorta* and *P*. *taiwanensis*, *psaM* was located in the SSC region, whereas in *P*. *massoniana*, it was located at the J_SA_ border (Fig 4). Similarly, in *P*. *taeda*, *P*. *contorta*, *P*. *tabuliformis*, *P*. *massoniana*, and *P*. *taiwanensis*, *psbB* was located in the LSC region at 478, 477, 505, 526, and 843 bp away from the J_LA_ border, respectively.

Large IRs play a significant role in stabilizing and maintaining the conserved structure of the cp genomes [94]. Various studies have reported that during the evolutionary process of angiosperms, a copy of an IR was lost, particularly in the subfamily Papilionoideae [95–97], and rearrangement in the chloroplast genome was observed because of IR loss in these genomes as compared to cp genomes with normal IRs [94]. Similarly, in gymnosperms, complete IRs were lost in conifers, particularly in cupressophytes and Pinaceae cp genomes, and greater rearrangement was observed in these genomes compared to in higher plants [33]. The remaining IR parts in various Pinaceae member and cupressophyte cp genomes were shown to differ, suggesting that these two conifer clades lost their large IRs independently during evolution from a common ancestor [78, 98]. Previously, it was reported that specific repeats in Pinaceae replaced the reduced IRs [99]. Compared to other conifers, a greater number of rearrangements occurred in *Pseudotsuga menziesii* and *P*. *radiate* cp genomes because of the lack of a large IR in these cp genomes [33]. Therefore, variation in the genome structure between *P*. *taeda* and related terrestrial plants, such as *C*. *japonica*, suggest that an IR is essential for structural stability of the cp genome.

### Repeat analysis

Repeat analysis of the *P*. *taeda* cp genome revealed six palindromic repeats, 34 forward repeats, and 22 tandem repeats (S1 Fig and Table 6). Among these, three forward repeats were 45–59 bp in length, with 14 tandem repeats of 15–29 bp in length (S1 Fig). Additionally, two palindromic repeats were 75–89 bp and four repeats were >90 bp (S1 Fig). Overall, 62 repeats were found in the *P*. *taeda* cp genome. Among tandem repeats, 12 repeats were in coding regions, eight repeats in intergenic regions, one repeat extending from an intergenic region into a coding region, and one repeat in the *petB* intron region (Table 7). The length of tandem repeats in these regions varied between eight and 14, and up to 10 repeat units were present. Various numbers of repeats have been identified in conifer cp genomes [100, 101] and the mechanisms implicit in the origin of these tandem repeats remain unclear. Nevertheless, they are known to be associated with chloroplast DNA rearrangement [102], gene expansion [100, 101], and gene duplication [103]. Previous reports suggested that repeat sequences, which play a role in genome rearrangement, are very helpful in phylogenetic studies [74, 104]. Furthermore, analyses of different cp genomes revealed that repeat sequences are important causes of indels and substitutions [101]. Sequence variation and cp genome re-arrangement occurs because of the slipped strand mis-pairing and improper recombination of repeat sequences [104–106]. The presence of such repeats shows that the locus is an important hotspot for cp genome re-configuration [74, 107]. In addition, such repeats contain crucial information for developing genetic markers for phylogenetic and population studies [74].

**Table 6:** Repeat sequences in the *Pinus taeda* chloroplast genome.

Repeat type	Repeat size	Repeat Position 1	Repeat location 1	Repeat Position 2	Repeat location 2
P	830	8692	*psbl*-*psbM*-*ycf12*	51,779	*ycf12*-*psbM*
P	399	66,445	*psbA-atpF*	121,132	IGS
P	304	50,503	IGS	120,845	IGS
P	277	50,530	IGS	120,845	IGS
P	86	0	*psbA*	66,359	*psbA*
P	79	9017	IGS	52,205	*psbM*-IGS
F	800	175	*psbA*	1815	IGS
F	376	109,649	*ycf2*	120,134	*ycf2*
F	288	50,861	IGS	84,618	IGS
F	284	50,843	IGS	84,600	IGS
F	275	50,825	IGS	84,582	IGS
F	247	51,131	*rps4*	70,403	*rps4*
F	185	50,964	IGS	84,721	IGS
F	171	51,207	*rps4*	70,479	*rps4*
F	165	100,638	*ycf1*	100,659	*ycf1*
F	124	101,059	IGS-*ycf1*	101,068	IGS-*ycf1*
F	97	9677	IGS	30,444	IGS
F	97	101,059	IGS-*ycf1*	101,113	IGS-*ycf1*
F	85	9737	IGS	30,504	IGS
F	70	100,733	*ycf1*	100,754	*ycf1*
F	79	9017	IGS	52,205	psbM
F	73	9701	IGS	30,468	IGS
F	71	100,638	*ycf1*	100,701	*ycf1*
F	70	100,712	*ycf1*	100,754	IGS
F	70	101,059	IGS-*ycf1*	101,122	*ycf1*
F	70	101,086	*ycf1*	101,140	*ycf1*
F	62	93,524	IGS	93,579	IGS
F	69	115,329	*ycf2*	115,395	ycf2
F	71	9777	*ycf1*	30,544	IGS
F	71	101,086	*ycf1*	101,149	*ycf1*
F	70	101,077	*ycf1*	101,140	*ycf1*
F	69	9714	IGS	30,481	IGS
F	58	71,811	IGS	71,831	IGS
F	67	101,149	*ycf1*	101,167	*ycf1*
F	61	101,059	*ycf1*	101,131	*ycf1*
F	64	101,057	*ycf1*	101,138	*ycf1*
F	63	101,057	*ycf1*	101,147	*ycf1*
F	59	101,043	*ycf1*	101,133	*ycf1*
F	55	100,895	ycf1 intron	100,976	*ycf1* intron
F	61	101,068	*ycf1*	101,149	*ycf1*

**Table 7:** Tandem repeat sequences in the *Pinus taeda* chloroplast genome.

Serial No	Indices	Repeat Length	Size of repeat unit × Copy number	A	C	G	T	Location
1	9274–9310	36	2 × 18	16	16	16	50	*PsaM/ycf12* (IGS)
2	15,199–15,235	36	2 × 18	44	8	23	23	*atpI* (CDS)
3	20,648–20,678	30	2 × 15	50	10	20	20	*rpoC2* (CDS)
4	28,466–28,534	68	2 × 34	30	24	12	33	*petN/psbM* (IGS)
5	31,275–31,313	38	2 × 19	23	13	36	26	*clpP*/IGS
6	33,103–33,166	63	3 × 21	29	16	19	33	*rps18* (CDS)
7	43,597–43,625	28	2 × 14	46	0	10	43	*accD/rbcL* (IGS)
8	43,615–43,659	44	2 × 22	40	12	8	38	*accD/rbcL* (IGS)
9	45,578–45,620	42	2 × 21	31	2	24	41	*rbcL/atpB* (IGS)
10	51,993–52,029	36	2 × 18	50	16	16	16	*ycf12/psbM* (IGS)
11	56,031–56,069	38	2 × 19	18	12	12	57	*petB* (intron)
12	93,544–93,631	87	3 × 29	37	16	10	35	*ycf68/chlL* (IGS)
13	93,525–93,635	110	2 × 55	35	15	11	36	*ycf68/chlL* (IGS)
14	97,002–97,056	54	2 × 27	28	20	24	26	*ycf1*(CDS)
15	100,583–100,631	48	2 × 24	54	9	18	16	*ycf1*(CDS)
16	100,639–100,828	189	9 × 21	45	9	28	16	*ycf1*(CDS)
17	100,827–101,025	198	6 × 33	31	1	43	23	*ycf1*(CDS)
18	100,866–101,016	150	10 × 15	30	1	44	23	*ycf1*(CDS)
19	100,827–101,953	126	2 × 63	31	1	43	23	*ycf1*(CDS)
20	100,823–101,985	162	2 × 81	32	2	42	22	*ycf1*(CDS)
21	100,939–101,047	108	2 × 54	34	4	38	22	*ycf1*(CDS)
22	115,330–115,452	122	2 × 66	21	22	11	45	*ycf2* (CDS)

### SSR analysis

**Fig 5:**
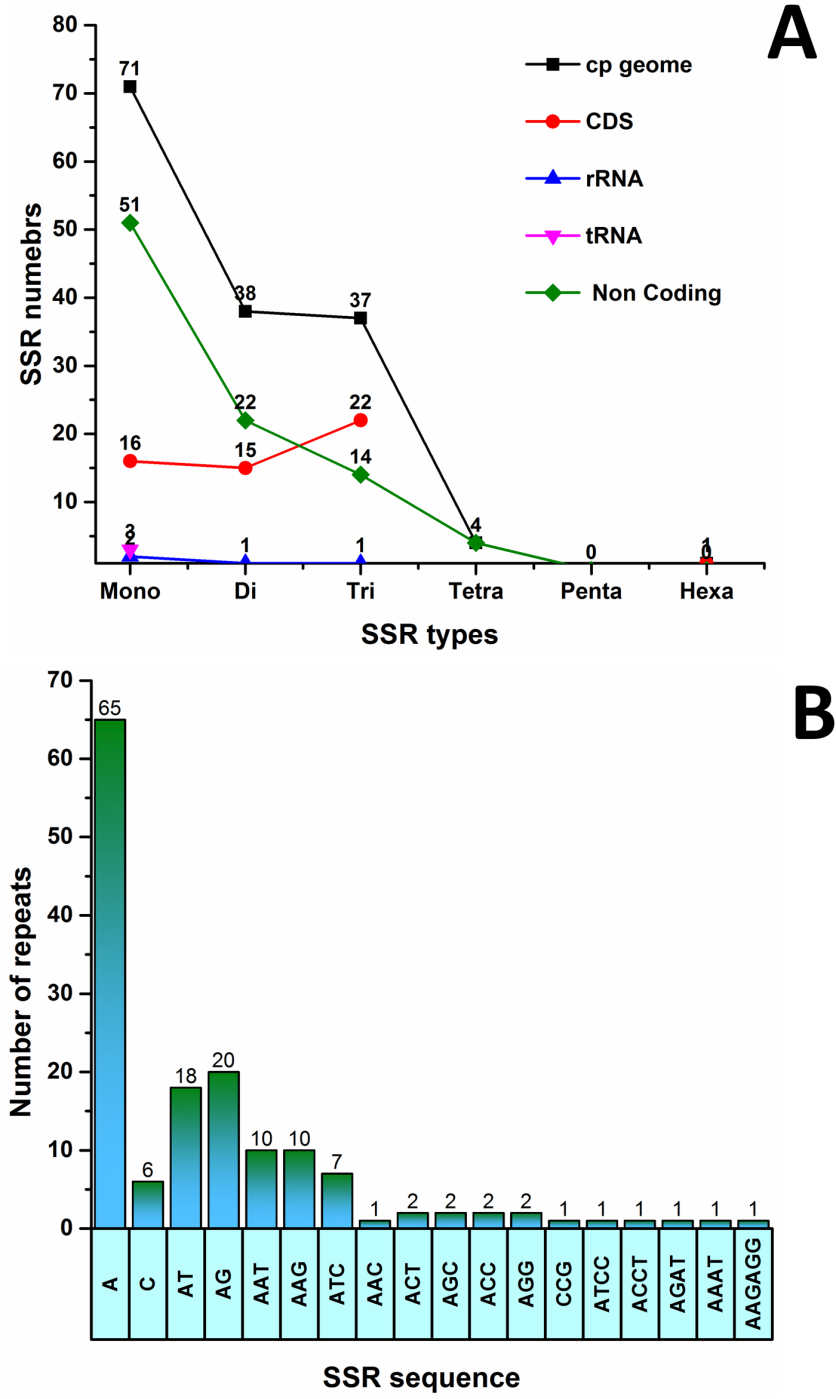
Analysis of simple sequence repeat (SSR) in the *Pinus taeda* plastid genome. **A**, Number of SSR types in complete genome, coding, and non-coding regions; **B**, Frequency of identified SSR motifs in different repeat class types.

SSRs are repeating sequences of typically 1–6 bp that are distributed throughout the genome. SSRs generally have a high mutation rate compared to neutral DNA regions because of slipped-strand mispairing. Because these short repeats are uniparentally inherited and haploid, they can be used as molecular markers in genetic studies analyzing population structures [108, 109]. In this study, we detected perfect SSRs in the *P*. *taeda* cp genome (Fig 5). Specific attributes were set for the analysis because SSRs (10 bp or longer) are exposed to slipped strand mis-pairing, the main mechanism of SSR polymorphisms [110–112]. A total of 151 perfect microsatellites were found in the *P*. *taeda* cp genome (Fig 5). Most (71) SSRs in this cp genome possessed a mononucleotide repeat motif. Dinucleotide SSRs were the second most common repeat motif (Fig 5B). Using our search criterion, four tetranucleotide SSRs and one hexanucleotide SSR were detected in the *P*. *taeda* cp genome (Fig 5A). In *P*. *taeda*, most mononucleotide SSRs were A (92.5%) and C (8.45%) motifs, with most dinucleotide SSRs being A/T (47.3%) and A/G (52.63%) motifs (Fig 5B and Table 8). Approximately 59.60% of SSRs were in non-coding regions, approximately 2.64% were present in rRNA sequences, and 1.98% were in tRNA genes (Fig 5A). These results are similar to those of previous reports showing that SSRs were unevenly distributed in cp genomes, and these findings may provide more information for selecting effective molecular markers for detecting intra- and interspecific polymorphisms [113–116]. Furthermore, analysis of various gymnosperm cp genomes revealed that most mononucleotides and dinucleotides are composed of A and T, which may contribute to bias in base composition, which is consistent with other cp genomes [117–119]. For SSR identification, although different criteria and algorithms were used, their distribution and characteristics were similar to the cp genomes of conifers [71, 119], 30 asterid [72], and 14 monocot [112]. Our findings were comparable to those of previous reports in which SSRs in cp genomes were found to be largely composed of polythymine (polyT) or polyadenine (polyA) repeats, and infrequently contained tandem cytosine (C) and guanine (G) repeats [118, 120]. Therefore, these SSRs contributed to the A-T richness of the *P*. *taeda* cp genome, which was also previously observed in the cp genomes of plant species [43, 71, 120]. The SSRs identified in the cp genome of *P*. *taeda* can be evaluated for polymorphisms at the intra-specific levels and used as markers for evaluating the genetic diversity of wild populations of plants from the Pinaceae family.

**Table 8:** Simple sequence repeats (SSRs) in the *Pinus taeda* chloroplast genome.

Unit	Length	No	SSR start
**A**	15	2	1375, 28,440
	14	3	68,741, 72,734, 106,240
	12	2	10,316, 110,251
	11	4	10,755, 26,980, 109,368, 11,873
	10	8	16,119, 22,252, 48,967, 83,427, 86,798, 88,062, 102,308, 111,412
	9	15	40,699, 41,827, 45,769, 70,952, 80,498, 80,744, 95,259, 102,053, 108,265, 110,985, 112,374, 113,688, 117,432, 119,716, 120,740
	8	31	4819, 10,738, 10,950, 16,110, 17,113, 30,189, 30,427, 30,701, 31,373, 33,345, 38,678, 41,893, 50,753, 51,485, 52622, 55,355, 56,042, 63,021, 64,394, 64,437, 92,458, 94,554, 95,822, 97,307, 103,868, 108,971, 114,282, 117065, 118885, 119,819, 120,893
**C**	9	4	16,101, 22,497, 71,353, 105,552
	8	2	31,381, 120,721
**AT**	13	1	41,344
	10	4	26,392, 96,162, 104,388, 113,787
	9	6	19,814, 24,397, 34,072, 42,422, 48,777, 74,253
	8	7	19,352, 19,904, 80,532, 83,639, 99,803, 105,218, 110,933
**AG**	9	10	8774, 22,311, 26,631, 47,568, 51,573, 52,520, 65,195,79,220, 80,699, 106,488,
	8	10	14,675, 22,384, 30,793, 42,926, 51,556, 69,139, 75,721, 83,721, 90,777, 91,093
**AAT**	11	1	78,353
	10	1	42,354
	9	8	13,934, 49,935, 65,369, 66,308, 71,749, 94,150, 98,727, 109,563
**AAG**	10	5	3167, 22,135, 106,110, 108,709, 120,693
	9	5	28,380, 79,051, 79,226, 81,004, 100,527
**ATC**	10	1	77,667
	9	6	2957, 16,215, 21,127, 75,445, 77,964, 111,780
**AAC**	9	1	32,982
**ACT**	9	2	43,692, 94,864
**AGC**	9	2	43,798, 89,223
**ACC**	9	2	54,293, 94,538
**AGG**	9	2	60,538, 80,037
**CCG**	9	1	
**ATCC**	17	1	48,863
**ACCT**	14	1	90,739
**AGAT**	13	1	51,753
**AAAT**	12	1	42,147
**AAGAGG**	23	1	117,038

### Phylogenetic analysis

In plants, the cp genome is a valuable resource for exploring intra- and interspecific evolutionary histories [121–127]. Compared to nuclear genomes in chloroplasts, the uniparental inheritance (for exceptions, see [122, 128]) is systematically striking because a single, independent genealogical history can be readily obtained for developing hypotheses [129–131]. Moreover, in some land plants (a few flowering plant lineages and conifers), the chloroplast is paternally inherited and independent of the nuclear and mitochondrial genome [132].

Recently, cp genomes have shown significant power in phylogenetic, evolution, and molecular systematics studies. During the last decade, various analyses have revealed the phylogenetic relationships at deep nodes based on comparisons of multiple protein coding genes, intergenic spacers [133, 134], and complete genome sequences in chloroplast genomes [135] that have enhanced our understanding of the evolutionary relationships among angiosperms and gymnosperms. According to the most recent classification, the genus *Pinus* is comprised of approximately 110 species and is shared by two subgenera, *Strobus* and *Pinus*, which are divided into further sections [136]. Furthermore, some evolutionary hypotheses suggest that the subgenera *Strobus* and *Pinus* originated from the Eocene [137, 138], whereas others indicated these subgenera were already present during the Cretaceous [138–140]. The *Pinus* subgenus has undergone significant distributional as well as environmental changes during their evolution, such as moving multiple times between America and Eurasia [140]. Chloroplast DNA polymorphisms in *P*. *taeda* have been used in numerous studies to assess paternal inheritance lineage and cytoplasmic diversity [141–146]. Continued efforts have expanded our ability to differentiate and understand the genomic structure and phylogenetic relationships of *Pinus* species [147]. The phylogeny and taxonomy of *Pinus* species have largely relied on chloroplast markers [140, 148, 149]. However, compared to nuclear genes, these markers are linked and offer independent information on species phylogeny. Previously, the phylogenetic study of pine based on multiple nuclear genes was reported by Syring et al. [150], where four low-copy nuclear loci were analyzed in 12 pine species and combined with internal transcribed spacers and chloroplast data. Various studies revealed that the addition of more genes increased the chance for improving the phylogenetic tree [151–153]. However, this does not resolve all phylogenetic problems [154, 155].

**Fig 6:**
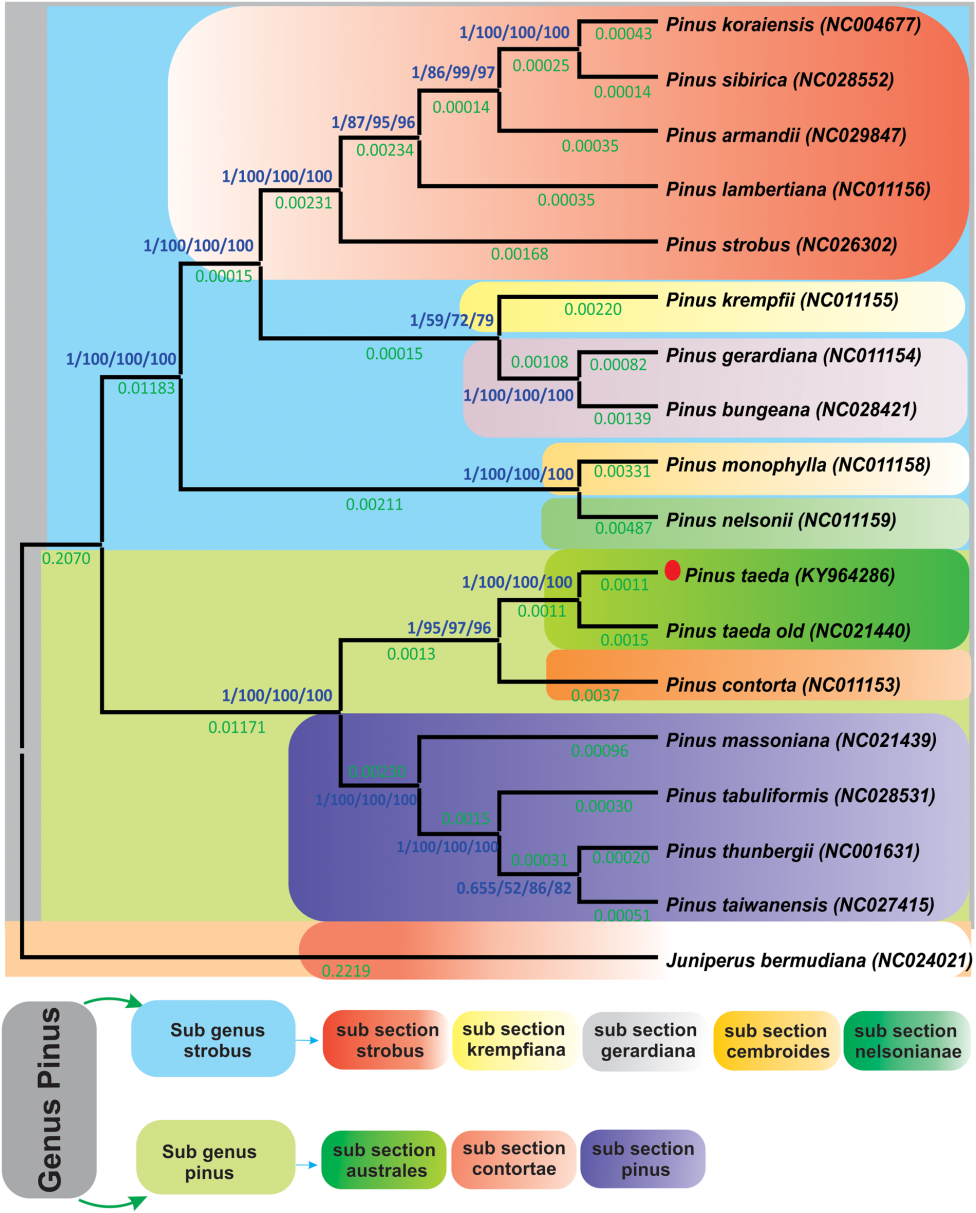
Phylogenetic trees of 15 *Pinus* species. The entire genome dataset was analyzed using four different methods: Bayesian inference (BI), maximum parsimony (MP), maximum likelihood (ML), and neighbor-joining (NJ). Numbers above the branches represent bootstrap values in the MP, ML, and NJ trees and posterior probabilities in the BI trees, whereas the number below the branches represents branch length. The red dot represents the position of *P*. *taeda* (KY964286).

Complete genome sequencing provides detailed insight into an organism [43, 66, 156]. In this study, the phylogenetic position of *P*. *taeda* within the *Pinus* genus was established by employing the complete cp genome and 60 shared genes of 16 species. Phylogenetic analyses using Bayesian inference, maximum parsimony, maximum likelihood, and neighbor-joining methods were performed. The phylogenetic analysis revealed that the complete dataset and 60 shared genes of *P*. *taeda* contained the same phylogenetic signals. In the datasets for the genome and 60 shared genes, *P*. *taeda* formed a single clade with *P*. *contorta* with high Bayesian interference and bootstrap support using the four different methods (Fig 6 and S2 Fig). Moreover, tree topology confirmed the relationship inferred from the phylogenetic work previously conducted based on cp genomes [89, 141, 157], in which *P*. *taeda* was genetically similar to *P*. *contorta*. These results revealed good agreement with classical taxonomy, where similar concordance was observed in the cp genome and mitochondrial genome-based reconstructions of *Pinus* phylogeny [136, 140]. Furthermore, these results are in broad agreement with previous results reported by Niu et al., where *P*. *taeda* formed a single clade with *P*. *contorta* based on pairwise non-synonymous substitution rates of orthologous transcripts [158]. Additionally, the results suggest that there is no conflict between the entire genome dataset and 60 shared genes in these cp genomes.

## Conclusion

The current study determined the complete genome sequence of the chloroplast from *P*. *taeda* (121,531 bp). The gene order and genome structure of *P*. *taeda* was similar to that of cp genomes of other *Pinus* species. Furthermore, the distribution and location of repeat sequences were determined, and average pairwise sequence divergences among cp genomes of related species were identified. SSR, SNP, and phylogenetic analyses were performed on 16 *Pinus* species cp genomes. No major structural rearrangement of *Pinus* species cp genomes was observed. Phylogenetic analyses revealed that the dataset based on 60 shared genes and that of the entire genome generated trees with the same topologies regarding the placement of *P*. *taeda*. Such investigations are an essential source of important information on the complete cp genome of *P*. *taeda* and related species, which can be used to facilitate biological study, identify species, and clarify taxonomic questions.

## Supporting information

S1 Table Primers used for gap closing and sequencing verification in *Pinus taeda*. (DOCX)

S2 Table Indel and SNP analysis of plastid genomes from *Pinus taeda* and 15 other *Pinus* species. (XLSX)

S3 Table Average pairwise distance of plastid sequences from *Pinus taeda* and 15 other *Pinus* species. (XLS)

S1 Fig Analysis of repeated sequences in *Pinus taeda* plastid genome. Total forward, tandem, and palindromic repeat sequences in the genome and their length distributions.
(TIF)

S2 Fig Phylogenetic trees were constructed for 15 species in the genus *Pinus* using different methods and the Bayesian tree is shown for the entire genome sequence. Data for 60 shared genes were used with four different methods: Bayesian inference (BI), maximum parsimony (MP), maximum likelihood (ML), and neighbor-joining (NJ). Numbers above the branches represent bootstrap values in the MP, ML, and NJ trees and posterior probabilities in the BI trees. The red dot represents the position of *P*. *taeda* (KY964286).
(TIF)
