# Supplementary material for: Complete chloroplast genome sequence and comparative analysis of loblolly pine (Pinus taeda L.) with related species
Source: PLoS One. 2018 Mar 29;13(3):e0192966. doi: 10.1371/journal.pone.0192966 (PMC5875761; doi:10.1371/journal.pone.0192966)
Supplement: S1 Table — (DOCX) [file pone.0192966.s001.docx]

| Number | Primers | Sequence | Size | Information |
| --- | --- | --- | --- | --- |
| 1 | 6087-F | AAACGGTCTCTCCAACGCAT | 514 bp | Gap Closing |
|  | 6300-R | ATGAGGCGGACCTTGGAAAG |  |  |
| 2 | 49766-F | TGGCATGTTTGGGACGATGA | 1023bp | Gap Closing |
|  | 50763-R | GCGGCTCTTTCCAAAAGACG |  |  |
| 3 | 105162-F | CCCACCCATAGAAGGAGGGA | 845bp | Gap Closing |
|  | 106993-R | TATCGTGCGATTGGTCTCGG |  |  |
| 4 | 119752-F | CCCACCCATAGAAGGAGGGA | 767bp | Gap Closing |
|  | 119220-R | TATCGTGCGATTGGTCTCGG |  |  |

**S1 Table. Primers used for gap closing and sequencing verification in *P. taeda***
