# Supplementary figures and images for: Complete chloroplast genome sequence and comparative analysis of loblolly pine (Pinus taeda L.) with related species
Source: PLoS One. 2018 Mar 29;13(3):e0192966. doi: 10.1371/journal.pone.0192966 (PMC5875761; doi:10.1371/journal.pone.0192966)

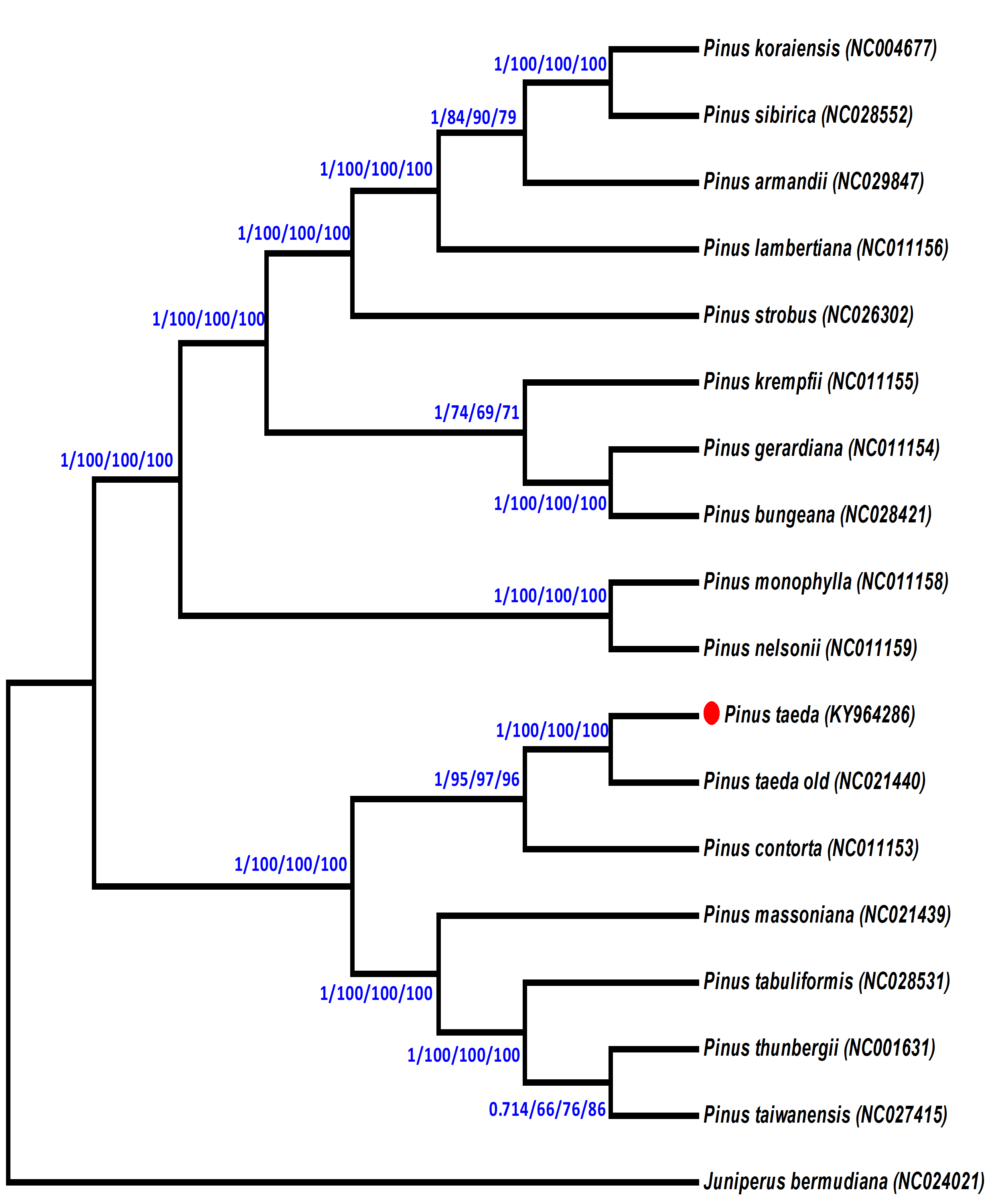

Supplement: S2 Fig — Data for 60 shared genes were used with four different methods: Bayesian inference (BI), maximum parsimony (MP), maximum likelihood (ML), and neighbor-joining (NJ). Numbers above the branches represent bootstrap values in the MP, ML, and NJ trees and posterior probabilities in the BI trees. The red dot represents the position of P. taeda (KY964286). (TIF) [file pone.0192966.s005.tif]
